# Alterations in Corticocortical Vestibular Network Functional Connectivity Are Associated with Decreased Balance Ability in Elderly Individuals with Mild Cognitive Impairment

**DOI:** 10.3390/brainsci13010063

**Published:** 2022-12-29

**Authors:** Rui Xia, Jinxin Ren, Xingjie Li, Jun Liu, Yalan Dai, Yuxing Kuang, Zhuguo Wu, Shangjie Chen

**Affiliations:** 1 Shenzhen Bao’an Clinical Medical School, Guangdong Medical University, Shenzhen 518100, China; 2 The Second School of Clinical Medicine, Guangdong Medical University, Dongguan 523808, China

**Keywords:** mild cognitive impairment, fall, corticocortical vestibular network, balance, functional connection

## Abstract

The corticocortical vestibular network (CVN) plays an important role in maintaining balance and stability. In order to clarify the specific relationship between the CVN and the balance ability of patients with mild cognitive impairment (MCI), we recruited 30 MCI patients in the community. According to age and sex, they were 1:1 matched to 30 older adults with normal cognitive function. We evaluated balance ability and performed MRI scanning in the two groups of participants. We analyzed functional connectivity within the CVN based on the region of interest. Then, we performed a Pearson correlation analysis between the functional connection and the Berg Balance Scale scores. The research results show that compared with the control group, there were three pairs of functional connections (hMST_R–Premotor_R, PFcm_R–SMA_L, and hMST_L–VIP_R) that were significantly decreased in the CVNs of the MCI group (*p* < 0.05). Further correlation analysis showed that there was a significant positive correlation between hMST_R–Premotor_R functional connectivity and BBS score (r = 0.364, *p* = 0.004). The decline in balance ability and increase in fall risk in patients with MCI may be closely related to the change in the internal connection mode of the corticocortical vestibular network.

## 1. Introduction

Falls and fall-related injuries are common and serious problems for elderly populations. Adults over the age of 65 have the highest risk of falling. In total, 30% of adults over 65 years of age and 50% of adults over 80 years of age fall at least once per year [1,2,3]. Falls are caused by the disparity between a person’s physical strength and their environment or the direct needs of ongoing activities. Every fall has the potential to change one’s life and may lead to chronic disability, assisted living, or even death. Even if the fall does not cause serious injury, it can also cause a fear of falling, which will lead to limited activity and increase the risk of future falls [4,5].

Mild cognitive impairment (MCI) is an early stage of Alzheimer’s disease; existing studies show that MCI is an important risk factor for instability and falls in the elderly [6]. Compared with elderly populations with normal cognitive function, the incidence of falls in MCI elderly individuals is higher [7]. Approximately 60% of elderly individuals with MCI fall at least once a year, which is twice the number of falls as those with normal cognitive function [1,8]. Patients with MCI are also more likely to experience traumatic and nonaccidental falls [9,10]. The incidence of hip fracture in elderly people with MCI after falling is three times higher than that in the general elderly population [11]. Therefore, it is important to prevent MCI patients from falling and to reduce the adverse consequences caused by falls.

Decline in balance ability is an important factor in the increased fall risk of MCI patients. A cross-sectional study involving 295 subjects showed that at as early as the stage of subjective cognitive decline, the balance ability of patients decreased significantly [12]. The fall risk index of patients with MCI was significantly increased, while Berg Balance Scale and gait index scores were significantly lower than those of healthy elderly people [13]. The maintenance of balance ability depends on the integration of vestibular inputs into the brain, which then adjust the efferent motor response [14]. The corticocortical vestibular network (CVN) is the core system that integrates vision, hearing and proprioception to maintain balance and stability [15]. Vestibule and other sensorimotor signals are primarily integrated in the dorsal striatum to control the movement of the body and limbs [16]. The central vestibular system receives inputs from two parallel information pathways. Regular signals transmit head rotation information while irregular signals only transmit information on high-frequency characteristics [17,18]. When the human body interacts with its environment, a multi-modal signal inflow is generated. The brain codes and estimates the stability of spontaneous motion and the motion of adjacent parts of the body to ensure the accurate control of behavior and posture in daily life. When the body is out of balance, the vestibular network sends out alarm signals, triggering defensive movement responses. At the level of neurophysiology, a decrease in gray matter in the vestibular network is closely related to a decrease in cognitive control ability and flexibility of posture, which reduces control over body posture balance [19,20]. Nerve conduction in the elderly is slower, and their vestibular network integration ability is weakened, their reaction time is prolonged, and they do not identify and avoid risks in a timely and effective manner [21]. The decline in control ability leads to an imbalance in balance ability, which significantly increases the risk of falls [22].

The change in functional connectivity of the CVN leads to a decline in balance ability and increases fall risk for MCI patients. However, previous studies have not studied the relationship between CVN connectivity and balance ability in patients with MCI in-depth, which has led to a lack of accurate targets in noninvasive brain stimulation techniques and other interventions. This study will use functional connectivity analysis based on the region of interest to observe the relationship between the functional connectivity of the CVN and balance ability in patients with MCI, with the goal of providing therapeutic targets for further intervention to reduce fall risk in MCI patients.

## 2. Materials and Methods

### 2.1. Participants

In total, 30 patients with MCI and 30 older adults with normal cognition were recruited; they were matched for age, sex, and education level. We recruited participants in communities near Shenzhen Bao’an Clinical Medical School, Guangdong Medical University, through posters, WeChat, and phone calls. The participants were assessed by doctors with more than 5 years of experience, according to the inclusion and exclusion criteria. The inclusion criteria of the MCI group included: (1) met the MCI diagnostic criteria (Petersen, 2004) [23]; (2) over 60 years old; and (3) right handedness. The inclusion criteria of the normal cognition (NC) group included: (1) did not meet the MCI diagnostic criteria (Petersen, 2004) [23]; (2) over 60 years old; and (3) right handedness. The exclusion criteria included: (1) cerebral infarction, cerebral hemorrhage, brain tumor, or other brain diseases that may affect the results of magnetic resonance imaging; (2) metal implants in the body, such as dentures, pacemakers, or intramedullary nails; (3) drug use that affects cognitive function; (4) accompanying neuropsychiatric disease, preventing patients from completing the cognitive assessment and balance ability test; (5) patients with existing vestibular or other neurological issues; and (6) accompanying claustrophobia, preventing patients from completing the MRI examination. The Shenzhen Bao’an Clinical Medical School of the Guangdong Medical University Ethics Committee for Clinical Research approved this study. All participants provided written informed consent after receiving information about the study.

### 2.2. Balance Ability Assessment

All participants were asked to fill in the Chinese version of the Berg Balance Scale (BBS). The scale has 38 items, and each item is scored from 0 to 4 points. The higher the score, the better the balance ability. The intragroup reliability of the scale in evaluating healthy elderly people was 0.97, showing good reliability [24].

### 2.3. Functional Magnetic Resonance Imaging Acquisition

A 3.0 T Siemens Prisma MRI scanner (Siemens, Erlangen, Germany) was used to obtain the T1 and resting state functional data of all participants. Resting state scans were acquired with the participants’ eyes closed and an echo planar sequence with the following parameters: TE/TR = 30 ms/2000 ms, flip angle = 90°, FOV = 230 mm, slices = 37, slice thickness = 3.6 mm, and time points = 300. A high-resolution T1-weighted MPRAGE was obtained (TR = 2300 ms, TE = 2.27 ms, flip angle = 8°, 160 slices, and voxel size = 0.98 × 0.98 × 1.0 mm^3^).

### 2.4. Functional Network Construction

Preprocessing of the fMRI data was performed using Functional Connectivity Toolbox (CONN) pipeline version 21a (www.nitrc.org/projects/conn, accessed on 5 September 2022). The preprocessing included slice-timing correction, segmentation, normalization, smoothing (Gaussian kernel = 8 mm), and band-pass filtering (0.008 Hz to 0.08 Hz). The specific preprocessing details were the same as those in Fang’s research [25].

We performed a functional connectivity analysis of the corticocortical vestibular network based on the region of interest (ROI). The ROIs of this study came from 20 activated brain regions induced by Raiser through tasks, including bilateral Area 2v, Area 3av, the premotor area, the cingulate sulcus visual (CSv), and the ventral intraparietal area (VIP), among others [26]. The ROIs were defined as a sphere with a radius of 5 mm in the Montreal Neurological Institute (MNI) space; we used the CONN toolbox to calculate the measured value of the ROI-to-ROI functional connection. To compare the functional connectivity of the vestibular network between patients with MCI and the NC group, white matter, cerebrospinal fluid signal, age, sex, education level, and cognitive function were controlled as covariates. Significant connections were identified by a two-sample *t*-test calculated as voxel threshold (uncorrected *p*-value of <0.05 and a cluster threshold of *p*-FDR < 0.05).

### 2.5. Statistical Analysis

The data were analyzed using SPSS 27.0 (IBM Corp., Armonk, NY, USA) for comparison of the clinical characteristics of the participants, and the independent sample t-test or the Kruskal–Wallis test as a nonparametric test was used for continuous variables and the chi-square test was used for binary variables. A *p*-value of <0.05 indicated that there was a statistical difference between groups. To analyze the correlation between the FC and the balance ability, a Pearson correlation coefficient test was performed.

## 3. Results

### 3.1. Subject Characteristics

Sex, age, and education level were not significantly different between the MCI and the control groups (all *p* > 0.05). The MoCA score and BBS score in the MCI group were significantly lower than those in the normal cognitive control group (*p* < 0.001) (Table 1).

### 3.2. Function Connection Analysis Results

**Figure 1:**
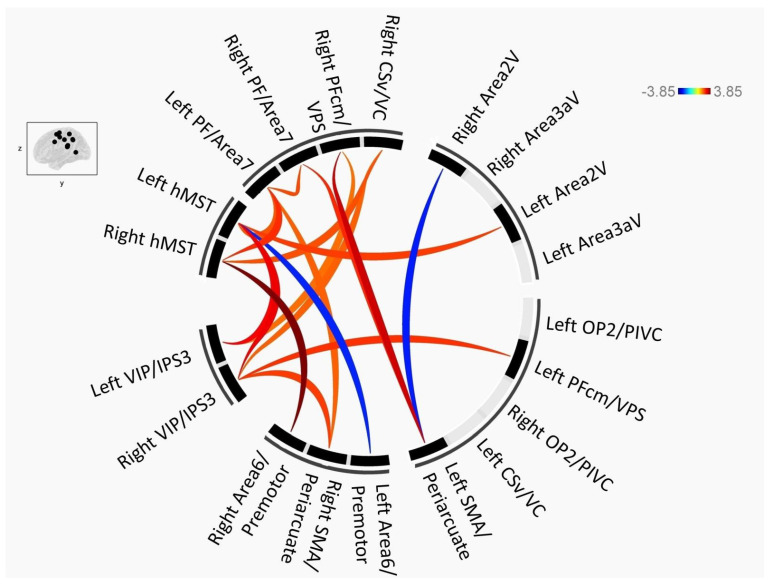
Connectogram depicting the differences in brain connectivity between the MCI group and the NC group. Voxel threshold: uncorrected *p* < 0.05 and cluster threshold: *p*-FDR < 0.05.

The functional connection between the ROIs of the cortical vestibular network in patients with MCI mainly decreased, and only the connection of two pairwise ROIs increased (Figure 1, where red indicates that the NC group was larger than the MCI and blue indicates that the NC group was smaller than the MCI). At the peak level, the MCI group had 2 pairwise functional connections that were enhanced and 15 pairwise function connections that were decreased compared with the NC group (*p* < 0.05). At the cluster level, after multiple comparison and correction (false discovery rate (FDR) corrected), three pairwise (hMST_R–premotor_R, PFcm_R–SMA_L, and hMST_L–VIP_R) functional connections in the MCI group were significantly lower than those in the NC group (*p* < 0.05) (Table 2).

### 3.3. Correlation Analysis

**Figure 2:**
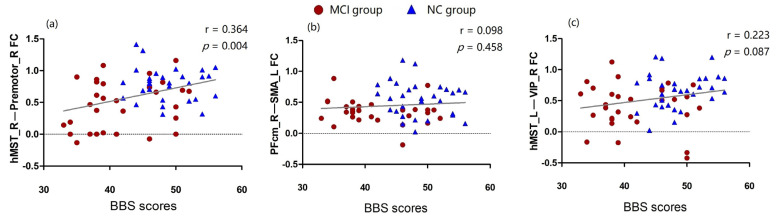
The linear correlation between the functional connection and the BBS scores. (**a**) correlation analysis of hMST_R–premotor_R functional connectivity and the BBS score(r = 0.364, *p* = 0.004); (**b**) correlation analysis of PFcm_R–SMA_L functional connectivity and the BBS score(r = 0.098, *p* = 0.458); (**c**) correlation analysis of hMST_L–VIP_R functional connectivity and the BBS score(r = 0.223, *p* = 0.087).

We extracted the z-values of three pairwise functional connections that retained statistical differences after the correction, and we conducted a correlation analysis with the participants’ BBS scores. There was a significant correlation between the hMST_R–premotor_R functional connectivity and the BBS score (r = 0.364 and *p* = 0.004) (Figure 2a). There was no significant correlation between the PFcm_R–SMA_L and hMST_L–VIP_R functional connectivity and the BBS score (*p* > 0.05) (Figure 2b,c).

## 4. Discussion

In this study, we compared the functional connections within the cortical vestibular network between patients with MCI and normal elderly people and found that there were significant differences among the three pairs of functional connections (hMST_R–premotor_R, PFcm_R–SMA_L, and hMST_L–VIP_R). Further correlation analysis showed that there was a significant positive correlation between the hMST_R–premotor_R functional connectivity and the BBS scores.

The CVN is composed of several distinct and separate areas which receive bilateral vestibular inputs from the vestibular nucleus, and they project directly down to the vestibular nucleus. Among them, parieto-insular vestibular cortex (PIVC) is regarded as the core of multisensory information processing [27,28]. However, in this study, the functional connection between bilateral PIVC and other components of CVN in MCI patients did not change significantly. This shows that the vestibular network damage of MCI patients has not yet affected PIVC. Nevertheless, proprioception, vision, hearing, and other signal input channels are not immune. The MST is thought to play an important role in heading perception, where the neurons in the MST are tuned to both head movement and vestibular signals [29,30,31,32]. The MST regulates the planning and execution of smooth pursuit eye movements, and its activities are affected by cognitive factors such as attention and working memory [33]. In addition, the MST has a causal effect on human self-motion perception and plays a key role in visually guided navigation [34]. The dorsal premotor cortex plays an essential role in visually guided goal-directed motor behavior [35]. In this study, we observed that the hMST_R–premotor_R functional connectivity in patients with MCI was significantly reduced. This may be the main manifestation of the impairment of patients’ vestibular network function, which has a negative impact on self-perception and exercise plans, thus leading to a decline in balance and an increase in the risk of falls for patients.

The VPS area shows vestibular-dominant heading tuning [36]. In the MST, the more extensive directional tuning of the putative interneurons is used for visual stimulation, while in VPS, the putative interneurons are more widely tuned to the vestibular state. It is assumed that the regional diversity of the tuning width difference between interneurons and pyramidal neurons is likely due to their dominant inputs [37]. The SMA is critical to many aspects of motor behavior, including motion preparation, motion initiation, and the selection, motion control, and monitoring of motion outcomes; at the same time, it plays an important role in auditory processing and participates in the sensorimotor process to guide auditory perception [38,39]. The decreased functional connectivity of the VPS–SMA may be due to the impaired auditory perception of patients with MCI, which leads to the weakening of multisensory integration and a decline in CVN function. The selectivity of the vestibular and visual flow signals lies between the MST and VPS, and as the VIP is an important brain region combining vestibular and visual flow signals [40,41,42], it may be proximal to the MST in terms of vestibular processing, but it is hierarchically similar to the MST in terms of optic flow processing [37]. It has also been found that the deactivation of the VIP area does not lead to behavioral defects and may not participate in visual heading discrimination [43]. Therefore, the weakening of the functional connection of the MST–VIP may be caused by the decreased ability of patients with MCI to process visual/vestibular signals.

In addition, we found that the connectivity of MCI patients’ periarcuate_L–area 2V_R increased. The periarcuate area plays an important role in coordinating eye and hand movements [44], and area 2V, located in the postcentral sulcus, can integrate optical flow clues in the processing of self-movement [45,46]. Enhancement of the periarcuate_L–area 2V_R functional connection may compensate for the motor coordination dysfunction caused by a reduction in visual signal input. Another interesting finding is that hMST_L–premotor_L functional connectivity is enhanced in patients with MCI, which is completely opposite to the result on the right side. Existing research cannot explain this result, and we speculate that it may be a partial compensation for the decline in contralateral functional connectivity.

The results of the correlation analysis showed that there was a significant positive correlation between the strength of hMST_R–premotor_R functional connection and BBS scores, but it has no significant correlation with MoCA scores. Previous evidence has suggested that the decline in balance function is not related to the severity of dementia or cognitive decline [47]. This study found similar results, indicating that cognitive function is a separate risk factor. The vestibular network is involved in two pathways that are critical to maintaining balance: the vestibulo-ocular reflex and the vestibulospinal reflex [48,49], which play key roles in controlling posture, gait, and fall risk [50,51]. On the basis of previous studies’ methods, this study used a more refined CVN template [26] to find the neuroimaging markers of impaired balance in patients with MCI. The results showed that the decreased functional connectivity of the hMST_R–premotor_R may be an early neuroimaging marker of impaired balance function in patients with MCI.

To summarize, the results of this study showed that functional connectivity within the CVN in patients with MCI is weakened, especially in the key brain regions of visual, auditory, and vestibular signal integration, and this is synchronous with a decrease in balance ability in patients with MCI. A decline in the functional connectivity of hMST_R and premotor_R may be an early sensitive marker for the decline in balance ability of MCI patients, which can be considered as an important target for future intervention.

This study had several limitations. Firstly, this was a preliminary study of a small sample, and it aimed to provide a more accurate examination for future interventions. Therefore, we did not perform more detailed assessments of vision, hearing, gait, dynamic balance ability, and other factors that affect falls. This may affect the stability of our results due to the interference of confounding factors; thus, we should collect information on other control variables in more detail in future research. Secondly, the corticocortical vestibular network seed points that we used were task-activated. However, this study used resting-state MRI data, which may have omitted some changes in the functional connectivity of the inactive brain regions in the resting state. Thirdly, this study was a case–control study, and the results of the correlation analysis cannot be used as the basis for causal inference. In future research, various factors that affect vestibular network function should be considered and evaluated in more detail. At the same time, tasks can be designed to activate the vestibular network or the sample size can be increased to improve the validity of the results. In addition, we can use noninvasive brain function regulation technology to stimulate the significantly different cortical vestibular network components found in this study in order to verify the value of these seed points in treatment. 

## 5. Conclusions

The decline in balance ability and the increase in fall risk among patients with MCI may be closely related to a change in the internal connection mode of the corticocortical vestibular network. In particular, the functional connectivity of hMST_R–premotor_R in patients with MCI is closely related to balance function score, which may be a sensitive marker for predicting falls.

## Figures and Tables

**Table 1:** Clinical characteristics of the participants.

Variable	MCI (N = 30)	NC (N = 30)	χ2/t Value	*p*-Value
Sex (M/F)	14/16	16/14	0.267	0.606
Age (years): mean ± SD	66.73 ± 5.20	67.50 ± 5.67	−0.546	0.587
Education level (years): Mean ± SD	9.53 ± 2.80	9.40 ± 2.58	0.192	0.849
MoCA (scores): mean ± SD	21.13 ± 2.46	27.60 ± 1.28	−12.782	<0.001
BBS (scores): mean ± SD	41.70 ± 5.93	48.67 ± 4.16	−5.269	<0.001

MCI, mild cognitive impairment; NC, normal cognitive control; MoCA, Montreal Cognitive Assessment; BBS, Berg Balance Scale; M, male; F, female; SD, standard deviation.

**Table 2:** Resting state pairwise connections in the MCI group compared to those of the NC group.

Compare	Analysis Unit		T	*p*-unc	*p*-FDR
	Seed	Target			
NC < MCI	Left periarcuate/SMA	Right Area 2V	−2.68	0.009524	0.079966
	Left hMST	Left Area 6/premotor	−2.61	0.011444	0.072479
NC > MCI	Right hMST	Right Area 6/premotor	3.85	0.000300	0.005707 *
	Right hMST	Left PF/area 7	2.57	0.012911	0.122658
	Right hMST	Right CSv	2.28	0.026072	0.165124
	Right PFcm/VPS	Left periarcuate/SMA	3.26	0.001883	0.035785 *
	Right PFcm/VPS	Left VIP	2.05	0.044867	0.275219
	Left periarcuate/SMA	Right PF/area 7	2.57	0.012626	0.079823
	Left PF/area 7	Right PF/area 7	2.36	0.021922	0.164857
	Left PF/area 7	Left hMST	2.26	0.027363	0.103979
	Left PF/area 7	Right periarcuate/SMA	2.16	0.034707	0.164876
	Left hMST	Right VIP	3.00	0.003958	0.048749 *
	Left hMST	Left VIP	2.91	0.005131	0.097498
	Left hMST	Left area 2V	2.43	0.018107	0.086009
	Right VIP	Left PFcm/VPS	2.52	0.014564	0.113398
	Right VIP	Right periarcuate/SMA	2.44	0.017905	0.125867
	Right VIP	Right CSv	2.16	0.034913	0.165835

MCI, mild cognitive impairment; NC, normal cognitive control; *p*-unc, *p*-value at peak level; *p*-FDR, *p*-value at cluster level; FDR, corrected false discovery rate; * *p* < 0.05; SMA, supplementary motor area; hMST, human medial superior temporal area; PF, prefrontal cortex; CSv, cingulate sulcus visual; PFcm, medial prefrontal cortex; VPS, visual posterior sylvian area; VIP, ventral intraparietal area; R, right; L, left.

## Data Availability

The data that support the findings of this study are available from the corresponding author upon reasonable request.

## Abbreviations

Abbreviations	Full Name
MCI	Mild Cognitive Impairment
NC	Normal Cognitive Control
CVN	Corticocortical Vestibular Network
BBS	Berg Balance Scale
MoCA	Montreal Cognitive Assessment
MRI	Magnetic Resonance Imaging
ROI	Region Of Interest
CSv	Cingulate Sulcus Visual
VIP	Ventral Intraparietal Area
MNI	Montreal Neurological Institute
FDR	False Discovery Rate Corrected
SMA	Supplementary Motor Area
hMST	Human Medial Superior Temporal Area
PF	Prefrontal Cortex
PFcm	Medial Prefrontal Cortex
VPS	Visual Posterior Sylvian Area
ECG	Electrocardiogram
SD	Standard Deviation
